# Ten SNPs May Affect Type 2 Diabetes Risk in Interaction with Prenatal Exposure to Chinese Famine

**DOI:** 10.3390/nu12123880

**Published:** 2020-12-18

**Authors:** Chao Song, Caicui Ding, Fan Yuan, Ganyu Feng, Yanning Ma, Ailing Liu

**Affiliations:** National Institute for Nutrition and Health, Chinese Center for Disease Control and Prevention, Beijing 100050, China; songchao@ninh.chinacdc.cn (C.S.); dingcc@ninh.chinacdc.cn (C.D.); yuanfan@ninh.chinacdc.cn (F.Y.); fenggy@ninh.chinacdc.cn (G.F.); mayn@ninh.chinacdc.cn (Y.M.)

**Keywords:** diabetes, famine, interaction, prenatal period

## Abstract

Increasing studies have demonstrated that gene and famine may interact on type 2 diabetes risk. The data derived from the cross-sectional 2010–2012 China National Nutrition and Health Survey (CNNHS) was examined to explore whether gene and famine interacted to influence type 2 diabetes risk. In total, 2216 subjects were involved. The subjects born in 1960 and 1961 were selected as the famine-exposed group, whereas subjects born in 1963 were selected as the unexposed group. A Mass Array system was used to detect the genotypes of 50 related single-nucleotide polymorphisms (SNPs). Interactions were found between prenatal exposure to famine and ten SNPs (rs10401969, rs10886471, rs10946398, rs1470579, rs2796441, rs340874, rs3794991, rs5015480, rs7961581, and rs9470794) on type 2 diabetes risk after adjustments. The stratified results showed that famine exposure exacerbated the effect of CILP2-rs10401969 to fasting serum insulin (FINS), GRK5-rs10886471 to fasting plasma glucose (FPG) and FINS, IGF2BP2-rs1470579 to FINS, TLE1-rs2796441 to impaired fasting glucose (IFG), PROX1-rs340874 to impaired glucose tolerance (IGT), GATAD2A-rs3794991 to FINS, TSPAN8/LGR5-rs7961581 to FPG, and ZFAND3-rs9470794 to IGT and FINS. Famine exposure weakened the effect of CDKAL1-rs10946398 to type 2 diabetes. Famine exposure weakened the effect of HHEX-rs5015480 to IFG, but exacerbated the effect of HHEX-rs5015480 to FINS. The present study suggests that ten SNPs may affect type 2 diabetes risk in interaction with prenatal exposure to Chinese famine.

## 1. Introduction

The occurrence of type 2 diabetes is not only influenced by the environment, but also by inherent cause. By associating regions of the genome with disease susceptibility, loci influencing type 2 diabetes risk have been identified [1]. Furthermore, convincing evidence has shown that genetic factors also play an important role in causing type 2 diabetes, and more than 100 loci have been confirmed to be contributable to type 2 diabetes risk in different ethnic populations, which promises to accelerate our understanding of disease pathology [2].

During the period of 1959–1961, Chinese people suffered the most severe famine in the world [3]. Some studies found that exposure to severe famine in the prenatal or postnatal period was associated with the development of type 2 diabetes in adulthood. Data from different periods of famine around the world have been utilized to explore the association of early life malnutrition and type 2 diabetes risk in adulthood, and the “famine effect” has been found in China and some foreign studies, including Asian, European, and African populations [4,5,6,7,8,9,10,11].

The individuals exposed to famine may involve adaptations to malnutrition, with fetal adaptations including reduced growth, small size at birth, or low birth weight [12]. One study assessed the interaction between birth weight and genetic susceptibility to type 2 diabetes in two independent prospective cohorts in the USA, and the data suggested that low birth weight and genetic susceptibility to obesity may affect adulthood risk of type 2 diabetes [13]. The latest research has found the existence of interactions between famine and some genes in the occurrence of type 2 diabetes, which means that some variants may influence susceptibility of type 2 diabetes amongst the population experiencing famine or malnutrition in early life, such as SIRT1, *PPAR*-γ*2* Pro12Ala, IGF2BP2, etc. [14,15,16]. The gene–environment interactions resulting from famine and increased type 2 diabetes risk have contributed to the epidemic of type 2 diabetes in China [17]. Thus, we used data from the China National Nutrition and Health Survey (CNNHS) 2010–2012 to explore whether there were some genetic variants which may affect type 2 diabetes risk with prenatal exposure to Chinese famine.

## 2. Materials and Methods

### 2.1. Data Resources

The CNNHS 2010–2012 was a national representative cross-sectional study which assessed the nutrition and health status of Chinese residents. The 2010–2012 survey covered all 31 provinces, autonomous regions, and municipalities throughout China (except for Taiwan, Hong Kong, and Macao). The country was divided into four strata (large cities, medium and small cities, ordinary rural areas, and poor rural areas), according to their characteristics of economy and social development, using the data from the China National Bureau of Statistics [18]. In this survey, subjects were recruited using a stratified multistage cluster and probability proportional to size sampling design, which was described in a previous study [19].

The Chinese famine lasted for three years, in 1959–1961. Therefore, we established our famine cohort: the subjects born in 1960–1961 were selected as the famine-exposed group, whereas subjects born in 1963 were selected as the unexposed group. The subjects in the two groups were 1:1 matched by gender and birth areas, with 1108 subjects in each group. Questionnaires were used to collect information on demographic characteristics. Blood samples were also collected from the subjects. The exclusion criteria were: unqualified blood sample; failure in DNA extraction; abnormal gene detect results; incomplete basic information; subjects suffered from liver/kidney/heart diseases/cancer; subjects had been diagnosed as type 2 diabetes and had changed their lifestyle.

The protocols of the 2010–2012 CNNHS and “Fetal origin hypothesis of diabetes: thrifty genotype hypothesis or thrifty phenotype” were both approved by the Ethical Committee of the National Institute for Nutrition and Health, Chinese Center for Disease Control and Prevention (No. 2013-018, No. 2013-010). Signed consent forms were obtained from all subjects.

### 2.2. Assessments of Variables

Information about demographic characteristics, dietary factors, smoking and drinking status, exercise data and anthropometric data were derived from the questionnaires. Self-reported education levels were classified as illiteracy to primary school, junior middle school, and senior high school or higher. Current economic status was assessed by the per capita annual income of households in 2011, and was divided into three levels: <20,000, 20,000–40,000 and >40,000 RMB. Smoking and drinking status was classified as “yes” or “no”.

A validated semi-quantitative food frequency questionnaire and 24 h recall method for the last three consecutive days (two weekdays and one weekend day) were used to collect data regarding dietary intake. In the present study, we only considered the intake of cereals and beans, and the intake of meat and poultry as confounders, as they have been found to be associated with type 2 diabetes [20,21]. The Chinese Dietary Guideline recommends that the reference intake of meat and poultry is between 40 g and 75 g, and the reference intake of cereals and beans is between 50 g and 150 g [22]. Thus, we assessed the intake according to the reference intake. The intake of meat and poultry was divided into three categories: low (<50 g/d), medium (from ≥50 to ≤150 g/d), and high (>150 g/d). Dietary intake of cereals and beans was divided into three categories: insufficient (<40 g/d), sufficient (from ≥40 to ≤75 g/d), and excessive (>75 g/d). Physical activity questionnaires were used to collect physical activity variables, such as whether exercise was taken or not, and total sedentary time (watching TV, using computers, playing video games, and reading) in the subjects’ leisure time. BMI was calculated as weight in kilograms divided by height in meters squared (kg/m^2^).

Fasting glucose was measured by collecting morning fasting venous blood samples. Then, the subjects without known diabetes were required to take 75 g oral glucose, and after two hours, venous blood samples were collected again to obtain 2-h plasma glucose. We used criteria proposed by the World Health Organization, the International Diabetes Federation, and the American Diabetes Association on diabetes mellitus [23,24,25,26]. Impaired fasting glucose (IFG) was defined as fasting plasma glucose (FPG) ≥6.1 and <7.0 mmol/L, 2-h plasma glucose <7.8 mmol/L. Impaired glucose tolerance (IGT) was defined as FPG <7.0 mmol/L and 2-h plasma glucose ≥7.8 and <11.1 mmol/L. Type 2 diabetes was defined as FPG ≥7.0 mmol/L and/or 2-h plasma glucose ≥11.0 mmol/L and/or a previous clinical diagnosis of type 2 diabetes. Fasting serum insulin (FINS) was measured by an Iodine [^125^I] Insulin Radioimmunoassay Kit.

### 2.3. Genotyping

According to the latest reports in the genome-wide association study and other studies, 61 related single-nucleotide polymorphisms (SNPs) were included in our study [27,28,29,30,31,32,33]. A Mass Array system (Agena, San Diego, USA) was used to detect the genotypes of 61 SNPs. No significant departures were detected from the Hardy–Weinberg equilibrium (HWE) among subjects without type 2 diabetes by using the chi-square test, which suggested the sample was representative (Supplementary Table S1). At the individual level, we removed the samples whose call rates were less than 50%. At the SNP level, we excluded the SNPs if their call rate was <80% and/or their *p*-value for HWE was <0.0001 in subjects without type 2 diabetes. Thus, 2216 subjects and 50 SNPs were ultimately included in the analysis.

### 2.4. Statistical Analysis

The statistical software package SAS version 9.4 (SAS Institute, Cary, NC, USA) was used for data analysis. A *p*-value < 0.05 was considered significant. Continuous variables were presented as mean ± SD or median (P25, P75) according to their distribution, and categorical variables were presented as frequency and percentage. Chi-square and *t*-tests were used for the comparison of differences between the exposed and unexposed groups. Interactions were tested by creating interaction terms for each genetic variant (coded 0, 1 for not carrying and carrying the risk allele respectively) with the exposed group (coded 0 and 1 for unexposed and exposed subjects, respectively). We tested the multiplicative interaction with famine exposure by using a likelihood ratio test comparing models with and without the cross-product term. Then, associations between SNPs and type 2 diabetes risk were performed according to fetal exposure to famine. General linear model regression was used to test the relationship between FPG, FINS, and SNPs, adjusting for covariates such as age, gender, education level, economic status, smoking, drinking, the intake of meat and poultry, the intake of cereals and beans, physical exercise, sedentary time, BMI, and family history of type 2 diabetes. Logistic regression was used to estimate the ORs for the risk of type 2 diabetes, IFG, and IGT after adjusting for the aforementioned covariates.

## 3. Results

A total of 2216 subjects were included in the current study, with an average age of 49.7 years. General characteristics of subjects between exposed and unexposed groups are shown in Table 1. There were group differences in age, education level and drinking.

Table 2 shows the interactions between genetic variants and prenatal exposure to famine as they influence type 2 diabetes risk. Interactions were found between prenatal exposure to famine and ten SNPs (rs10401969, rs10886471, rs10946398, rs1470579, rs2796441, rs340874, rs3794991, rs5015480, rs7961581 and rs9470794) and type 2 diabetes risk after adjustments for age, gender, education level, economic status, smoking, drinking, the intake of meat and poultry, the intake of cereals and beans, physical exercise, sedentary time, BMI, and family history of type 2 diabetes (*p* < 0.05).

Table 3 showed that FPG increased by 0.474 mmol/L among risk allele carriers (rs10886471) in the exposed group (*p* = 0.032), and FINS decreased by 2.996 mU/L among risk allele carriers in the unexposed subjects (*p* = 0.023). There was a significant association for rs10946398 with type 2 diabetes for risk allele carriers in the unexposed group (OR = 3.263, 95%CI: 1.584–6.724, *p* = 0.001). FINS increased by 1.427 mU/L among risk allele carriers (rs1470579) in the exposed subjects (*p* = 0.011). FINS increased by 1.725 mU/L among risk allele carriers (rs3794991) in the exposed group (*p* = 0.046). There was a significant association for rs5015480 with IFG (OR = 1.941, 95%CI: 1.119–3.366, *p* = 0.018) for risk allele carriers in the unexposed group, and FINS increased by 1.260 mU/L among risk allele carriers in the exposed group (*p* = 0.032). FPG increased by 0.171 mmol/L among risk allele carriers (rs7961581) in the exposed subjects (*p* = 0.042). There was a significant association of rs9470794 with IGT for risk allele carriers in the exposed group (OR = 7.902, 95%CI: 1.063–58.735, *p* = 0.043), and FINS increased by 2.105 mU/L among risk allele carriers in the exposed group (*p* = 0.018). In the exposed subjects, the risk allele carriers (rs10401969) tended to increase with FINS (*p* = 0.092), whereas this was not true in the unexposed subjects (*p* = 0.210). There was a borderline significant association between rs2796441 and IFG (OR = 0.587, 95%CI: 0.336–1.026), *p* = 0.061), as well as rs340874 and IGT (OR = 0.616, 95%CI: 0.352–1.077, *p* = 0.089) in the unexposed subjects.

## 4. Discussion

The present study indicates that CILP2-rs10401969, GRK5-rs10886471, CDKAL1-rs10946398, IGF2BP2-rs1470579, TLE1-rs2796441, PROX1-rs340874, GATAD2A-rs3794991, HHEX-rs5015480, TSPAN8/LGR5-rs7961581, and ZFAND3-rs9470794 showed nominally significant interactions with prenatal exposure to famine in type 2 diabetes risk.

The Chinese famine provides a unique opportunity to investigate the interactions of prenatal exposure to famine with type 2 diabetes and related measurements. The latest studies have found that prenatal exposure to famine interacted with some genes in influencing type 2 diabetes [14,15,16]. Thus, we investigated interactions of SNPs associated with type 2 diabetes in the Chinese population exposed to famine in utero. Our stratified results showed that famine exposure exacerbated the effect of CILP2-rs10401969 to FINS, GRK5-rs10886471 to FPG and FINS, IGF2BP2-rs1470579 to FINS, TLE1-rs2796441 to IFG, PROX1-rs340874 to IGT, GATAD2A-rs3794991 to FINS, TSPAN8/LGR5-rs7961581 to FPG, and ZFAND3-rs9470794 to IGT and FINS. Famine exposure weakened the effect of CDKAL1-rs10946398 to type 2 diabetes. Famine exposure weakened the effect of HHEX-rs5015480 to IFG, but exacerbated the effect of HHEX-rs5015480 to FINS. To our knowledge, the ten SNPs are the first found to interact with prenatal exposure to famine in type 2 diabetes risk.

The IGF2BP2 gene, which encodes the IGF2 mRNA binding protein2, is suggested to play a role in the regulation of insulin production and beta cell function, and IGF2BP2-rs4402960 showed an interaction with prenatal exposure to famine on glucose levels in the Dutch Famine Birth Cohort Study in Amsterdam [15]. Some studies explored the interaction of genes and fetal malnutrition or birth size/weight in type 2 diabetes risk (K121Q, HHEX, CDKN2A/2B, etc.) [12,34]. However, IGF2BP2-rs4402960 did not show an interaction between birth weight and the risk of developing type 2 diabetes in the Helsinki Birth Cohort Study [34]. Variants in CDKAL1 were associated with beta cell function and influenced insulin secretion. The Helsinki Birth Cohort Study investigated the interaction between birth weight and CDKAL1-rs7754840 on the risk of developing type 2 diabetes, and the results were negative [34]. CDKAL1-rs10946398 was previously reported to be associated with birth weight and type 2 diabetes [35], so it was possible that CDKAL1-rs10946398 influenced type 2 diabetes risk by affecting birth weight, or CDKAL1-rs10946398 indeed had an interaction with prenatal exposure to famine in type 2 diabetes risk, but such explanations are speculative and they still need to be replicated in different cohorts. HHEX was associated with impaired insulin release by influencing beta cell development, and HHEX-rs1111875 was found to have an interaction with low birth weight in type 2 diabetes in the Helsinki Birth Cohort Study, which indicated that low birth weight might affect the strength of the association of the variants with type 2 diabetes [34,36].

CILP2 encodes cartilage intermediate layer protein 2, GRK5 plays a crucial role in multiple G-protein-coupled receptors(GPCRs) and non-GPCR substrates which are either key regulators of glucose homeostasis or inflammation, TLE1 and GATAD2A are protein-coding genes, PROX1 influences insulin secretion by influencing beta cell development, TSPAN8/LGR5 seems to result in pancreatic beta cell dysfunction and influences insulin secretion, and the expression of ZFAND3 was found in mouse pancreatic islets with altered beta-cell function [2,29,31,36]. Previous researchers found that exposure to famine in utero or food restriction during gestation impaired and reduced glucose tolerance or decreased beta cell mass [16], which predisposed humans to type 2 diabetes in later life [37,38]. Most of these type 2 diabetes susceptibility genes were associated with the expression and/or function in beta cells and changed insulin secretion. Whether these SNPs involved in fetal development can influence type 2 diabetes in adulthood still needs to be replicated in later cohorts.

The present study has several advantages. It was the first time the interaction of so many SNPs and fetal exposure to Chinese famine in type 2 diabetes risk was examined. Moreover, our study utilized national representative data and provided a scenario to assess whether the variants influence the established association between prenatal exposure to famine and type 2 diabetes risk. We found several variants that showed interactions, although these variants still need to be confirmed in later studies. There were also some limitations which should be mentioned. Although we considered some lifestyle factors, other confounding factors such as the consumption of sugar-sweetened beverages, eggs, and fruits and vegetables were not considered in our study. Additionally, the mechanism of how these SNPs interact with prenatal famine on type 2 diabetes risk still remains unclear, and should be examined in the future.

## 5. Conclusions

Our study suggests that ten SNPs may be genetic factors influencing type 2 diabetes risk among famine-exposed subjects, which might synergistically impair the development and function of beta cells, increasing type 2 diabetes risk in adulthood.

## Figures and Tables

**Table 1:** Basic information of subjects.

Variables	Total	Unexposed	Exposed	*p*-Value
N	2216	1108	1108	
Age (years)	49.7 (48.7, 51.3)	48.8 (48.3, 49.4)	51.1 (50.3, 51.7)	<0.001 *
**Gender**				0.965
Male	879 (39.7%)	439 (39.6%)	440 (39.7%)	
Female	1337 (60.3%)	669 (60.4%)	668 (60.3%)	
**Areas**				1.000
Medium and small cities	720 (32.5%)	360 (32.5%)	360 (32.5%)	
Ordinary rural areas	1002 (45.2%)	501 (45.2%)	501 (45.2%)	
Poor rural areas	494 (22.3%)	247 (22.3%)	247 (22.3%)	
**Education level**				0.026 *
Illiterate to primary school	787 (35.5%)	369 (33.3%)	418 (37.7%)	
Junior middle school	951 (42.9%)	506 (45.7%)	445 (40.2%)	
Senior high school or higher	478 (21.6%)	233 (21.0%)	245 (22.1%)	
**Family’s economic level (RMB/Year/per capita)**				0.614
<20,000	1146 (51.7%)	565 (51.0%)	581 (52.4%)	
20,000–40,000	834 (37.6%)	420 (37.9%)	414 (37.4%)	
>40,000	157 (7.1%)	78 (7.0%)	79 (7.1%)	
Unknown	79 (3.6%)	45 (4.1%)	34 (3.1%)	
**Smoking**				0.493
No	1555 (70.2%)	789 (71.2%)	766 (69.1%)	
Yes	658 (29.7%)	318 (28.7%)	340 (30.7%)	
Unknown	3 (0.1%)	1 (0.1%)	2 (0.2%)	
**Drinking**				0.004 *
No	1472 (66.4%)	722 (65.2%)	750 (67.7%)	
Yes	742 (33.5%)	386 (34.8%)	356 (32.1%)	
Unknown	2 (0.1%)	0	2 (0.2%)	
**Intake of cereals and beans**				0.908
Insufficient	1452 (65.5%)	733 (66.2%)	719 (64.9%)	
Sufficient	185 (8.3%)	93 (8.4%)	92 (8.3%)	
Excessive	42 (1.9%)	20 (1.8%)	22 (2.0%)	
Unknown	537 (24.2%)	262 (23.7%)	275 (24.8%)	
**Intake of meat and poultry**				0.163
Low	692 (31.2%)	361 (32.6%)	331 (29.9%)	
Medium	382 (17.2%)	174 (15.7%)	208 (18.8%)	
High	605 (27.3%)	311 (28.1%)	294 (26.5%)	
Unknown	537 (24.2%)	262 (23.7%)	275 (24.8%)	
**Physical exercise**				0.192
No	2009 (90.7%)	995 (89.8%)	1014 (91.5%)	
Yes	192 (8.7%)	107 (9.7%)	85 (7.7%)	
Unknown	15 (0.7%)	6 (0.5%)	9 (0.8%)	
Sedentary time(h/d)	2.0 (2.0, 3.0)	2.0 (2.0, 3.0)	2.0 (2.0, 3.0)	0.196
**Family history of diabetes**				
No	2173 (98.1%)	1084 (97.8%)	1089 (98.3%)	0.441
Yes	43 (1.9%)	24 (2.2%)	19 (1.7%)	
BMI (kg/m^2^)	24.0 (21.9, 26.4)	24.1 (22.0, 26.4)	23.9 (21.8, 26.5)	0.708
FPG (mmol/L)	5.2 (4.7, 5.7)	5.1 (4.7, 5.6)	5.2 (4.7, 5.7)	0.425
**Diabetes**				0.427
No	2079 (93.8%)	1035 (93.4%)	1044 (94.2%)	
Yes	137 (6.2%)	73 (6.6%)	64 (5.8%)	
**IGT**				0.886
No	1960 (94.3%)	975 (94.2%)	985 (94.3%)	
Yes	119 (5.7%)	60 (5.8%)	59 (5.7%)	
**IFG**				0.824
No	1950 (93.8%)	972 (93.9%)	978 (93.7%)	
Yes	129 (6.2%)	63 (6.1%)	66 (6.3%)	
FINS (mU/L)	12.6 (9.3, 15.7)	12.7 (9.4, 15.6)	12.6 (9.2, 15.8)	0.870

Data are presented as mean ± SD for continuous variables and N (%) for categorical variables. * *p* < 0.05.

**Table 2:** Interactions of single-nucleotide polymorphisms (SNPs) with prenatal exposure to famine.

SNP	Loci	Diabetes	IGT	IFG	FPG	FINS
rs10401969	CILP2	0.145	0.763	0.545	0.094	0.046 *
rs10830963	MTNR1B	0.314	0.565	0.347	0.393	0.906
rs10842994	KLHDC5	0.903	0.701	0.888	0.736	0.916
rs10886471	GRK5	0.900	0.872	0.659	0.258	0.005 *
rs10906115	CDC123, CAMK1D	0.969	0.187	0.164	0.514	0.748
rs10946398	CDKAL1	0.005 *	0.442	0.935	0.238	0.400
rs11257655	CDC123	0.657	0.398	0.243	0.705	0.766
rs11634397	ZFAND6	0.337	0.987	0.936	0.399	0.513
rs12454712	BCL2	0.365	0.152	0.136	0.540	0.614
rs12970134	MC4R	0.549	0.211	0.286	0.248	0.416
rs13266634	SLC30A8	0.695	0.439	0.284	0.805	0.860
rs1470579	IGF2BP2	0.612	0.829	0.142	0.635	0.022 *
rs1535500	KCNK16	0.482	0.347	0.491	0.553	0.795
rs1552224	CENTD2	0.951	0.959	0.954	0.648	0.873
rs1558902	FTO	0.119	0.091	0.291	0.254	0.700
rs16861329	ST6GAL1	0.643	0.064	0.112	0.382	0.553
rs17584499	PTPRD	0.432	0.508	0.453	0.351	0.775
rs2028299	AP3S2	0.948	0.574	0.452	0.843	0.820
rs2191349	DGKB, TMEM195	0.284	0.579	0.139	0.860	0.319
rs243021	BCL11A	0.611	0.403	0.090	0.296	0.342
rs2796441	TLE1	0.412	0.172	0.039 *	0.709	0.322
rs2943641	IRS1	0.999	0.952	0.949	0.881	0.360
rs340874	PROX1	0.830	0.024 *	0.527	0.687	0.958
rs3794991	GATAD2A	0.720	0.353	0.456	0.966	0.018 *
rs3923113	GRB14	0.903	0.888	0.160	0.399	0.059
rs4430796	HNF1B	0.565	0.516	0.787	0.512	0.341
rs459193	ANKRD55	0.627	0.867	0.925	0.976	0.099
rs4607103	ADAMTS9	0.583	0.714	0.187	0.426	0.338
rs4607517	GCK	0.719	0.116	0.567	0.461	0.056
rs4858889	SCAP	0.383	0.980	0.824	0.765	0.845
rs5015480	HHEX	0.785	0.377	0.036 *	0.428	0.346
rs516946	ANK1	0.804	0.927	0.474	0.793	0.700
rs5215	KCNJ11	0.943	0.477	0.192	0.564	0.567
rs6815464	MAEA	0.820	0.194	0.081	0.286	0.395
rs7041847	GLIS3	0.265	0.193	0.088	0.053	0.299
rs7172432	C2CD4A, C2CD4B	0.133	0.344	0.381	0.522	0.588
rs7178572	HMG20A	0.108	0.946	0.998	0.711	0.274
rs7202877	BCAR1	0.917	0.881	0.852	0.762	0.817
rs7403531	RASGRP1	0.572	0.080	0.802	0.489	0.184
rs7593730	RBMS1, ITGB6	0.884	0.261	0.315	0.528	0.958
rs7612463	UBE2E2	0.967	0.882	0.146	0.176	0.291
rs780094	GCKR	0.566	0.869	0.277	0.589	0.178
rs7961581	TSPAN8, LGR5	0.073	0.642	0.163	0.017 *	0.944
rs8050136	FTO	0.102	0.458	0.360	0.282	0.867
rs8090011	LAMA1	0.114	0.679	0.499	0.177	0.851
rs831571	PSMD6	0.569	0.402	0.815	0.659	0.144
rs864745	JAZF1	0.453	0.283	0.800	0.188	0.939
rs896854	TP53INP1	0.184	0.747	0.452	0.280	0.191
rs9470794	ZFAND3	0.946	0.146	0.053	0.356	0.007 *
rs972283	KLF14	0.704	0.616	0.162	0.837	0.155

Data are presented as *p*-value; * *p* < 0.05.

**Table 3:** Associations between SNPs and diabetes risk according to prenatal exposure to famine.

SNP	Group	Risk Allele	Diabetes	*p*-Value	IGT	*p*-Value	IFG	*p*-Value	FPG	*p* Value	FINS	*p*-Value
			OR (95%CI)		OR (95%CI)		OR (95%CI)		β		β	
rs10401969	unexposed	0	1.00 (Ref)		1.00 (Ref)		1.00 (Ref)		1.00 (Ref)		1.00 (Ref)	
	unexposed	1	1.007 (0.496, 2.045)	0.985	1.070 (0.522, 2.196)	0.853	1.592 (0.807, 3.140)	0.180	0.123 (−0.062, 0.309)	0.193	−0.959 (−2.459, 0.541)	0.210
	exposed	0	1.00 (Ref)		1.00 (Ref)		1.00 (Ref)		1.00 (Ref)		1.00 (Ref)	
	exposed	1	0.502 (0.193, 1.306)	0.158	0.978 (0.456, 2.099)	0.955	1.048 (0.524, 2.095)	0.895	−0.106 (−0.325, 0.113)	0.345	1.314 (−0.214, 2.842)	0.092
rs10886471	unexposed	0	1.00 (Ref)		1.00 (Ref)		1.00 (Ref)		1.00 (Ref)		1.00 (Ref)	
	unexposed	1	1.677 (0.373, 7.538)	0.500	†		1.605 (0.370, 6.965)	0.527	0.201 (−0.124, 0.526)	0.225	−2.996 (−5.568, −0.424)	0.023 *
	exposed	0	1.00 (Ref)		1.00 (Ref)		1.00 (Ref)		1.00 (Ref)		1.00 (Ref)	
	exposed	1	1.361 (0.300, 6.178)	0.689	1.623 (0.210, 12.547)	0.643	2.754 (0.356, 21.301)	0.332	0.474 (0.040, 0.907)	0.032 *	2.498 (−0.112, 5.109)	0.061
rs10946398	unexposed	0	1.00 (Ref)		1.00 (Ref)		1.00 (Ref)		1.00 (Ref)		1.00 (Ref)	
	unexposed	1	3.263 (1.584, 6.724)	0.001 *	1.163 (0.654, 2.067)	0.608	1.157 (0.650, 2.058)	0.621	0.085 (−0.059, 0.228)	0.247	0.025 (−1.134, 1.184)	0.966
	exposed	0	1.00 (Ref)		1.00 (Ref)		1.00 (Ref)		1.00 (Ref)		1.00 (Ref)	
	exposed	1	0.830 (0.466, 1.478)	0.526	0.867 (0.481, 1.565)	0.636	1.167 (0.642, 2.122)	0.613	−0.050 (−0.223, 0.124)	0.574	−0.663 (−1.841, 0.515)	0.270
rs1470579	unexposed	0	1.00 (Ref)		1.00 (Ref)		1.00 (Ref)		1.00 (Ref)		1.00 (Ref)	
	unexposed	1	1.373 (0.798, 2.363)	0.253	1.062 (0.619, 1.821)	0.827	1.236 (0.719, 2.127)	0.443	0.039 (−0.098, 0.175)	0.578	−0.400 (−1.519, 0.720)	0.483
	exposed	0	1.00 (Ref)		1.00 (Ref)		1.00 (Ref)		1.00 (Ref)		1.00 (Ref)	
	exposed	1	0.990 (0.571, 1.715)	0.970	1.157 (0.659, 2.030)	0.611	0.703 (0.404, 1.225)	0.214	−0.024 (−0.186, 0.139)	0.774	1.427 (0.335, 2.518)	0.011 *
rs2796441	unexposed	0	1.00 (Ref)		1.00 (Ref)		1.00 (Ref)		1.00 (Ref)		1.00 (Ref)	
	unexposed	1	1.055 (0.599, 1.856)	0.854	1.346 (0.733, 2.473)	0.338	0.587 (0.336, 1.026)	0.061	−0.000 (−0.146, 0.145)	0.997	0.058 (−1.118, 1.234)	0.923
	exposed	0	1.00 (Ref)		1.00 (Ref)		1.00 (Ref)		1.00 (Ref)		1.00 (Ref)	
	exposed	1	0.740 (0.423, 1.295)	0.292	0.790 (0.444, 1.404)	0.421	1.376 (0.769, 2.460)	0.282	−0.025 (−0.195, 0.146)	0.778	−0.779 (−1.896, 0.339)	0.172
rs340874	unexposed	0	1.00 (Ref)		1.00 (Ref)		1.00 (Ref)		1.00 (Ref)		1.00 (Ref)	
	unexposed	1	0.989 (0.567, 1.726)	0.969	0.616 (0.352, 1.077)	0.089	0.900 (0.511, 1.586)	0.716	−0.036 (−0.182, 0.111)	0.634	−0.318 (−1.500, 0.863)	0.597
	exposed	0	1.00 (Ref)		1.00 (Ref)		1.00 (Ref)		1.00 (Ref)		1.00 (Ref)	
	exposed	1	0.812 (0.459, 1.434)	0.472	1.472 (0.799, 2.713)	0.215	0.696 (0.407, 1.190)	0.185	0.019 (−0.149, 0.188)	0.822	−0.269 (−1.284, 0.747)	0.604
rs3794991	unexposed	0	1.00 (Ref)		1.00 (Ref)		1.00 (Ref)		1.00 (Ref)		1.00 (Ref)	
	unexposed	1	0.606 (0.245, 1.499)	0.278	0.532 (0.187, 1.516)	0.237	0.909 (0.374, 2.208)	0.833	0.002 (−0.215, 0.219)	0.988	−1.378 (−3.168, 0.412)	0.131
	exposed	0	1.00 (Ref)		1.00 (Ref)		1.00 (Ref)		1.00 (Ref)		1.00 (Ref)	
	exposed	1	1.039 (0.424, 2.546)	0.934	0.973 (0.395, 2.397)	0.953	0.524 (0.184, 1.497)	0.228	0.033 (−0.224, 0.290)	0.803	1.725 (0.035, 3.416)	0.046 *
rs5015480	unexposed	0	1.00 (Ref)		1.00 (Ref)		1.00 (Ref)		1.00 (Ref)		1.00 (Ref)	
	unexposed	1	0.995 (0.551, 1.798)	0.988	1.030 (0.575, 1.844)	0.921	1.941 (1.119, 3.366)	0.018 *	0.045 (−0.099, 0.189)	0.540	0.369 (−0.803, 1.542)	0.536
	exposed	0	1.00 (Ref)		1.00 (Ref)		1.00 (Ref)		1.00 (Ref)		1.00 (Ref)	
	exposed	1	1.236 (0.699, 2.183)	0.466	0.665 (0.351, 1.262)	0.212	0.839 (0.460, 1.529)	0.566	−0.029 (−0.203, 0.144)	0.740	1.260 (0.108, 2.412)	0.032 *
rs7961581	unexposed	0	1.00 (Ref)		1.00 (Ref)		1.00 (Ref)		1.00 (Ref)		1.00 (Ref)	
	unexposed	1	0.625 (0.349, 1.119)	0.114	0.710 (0.405, 1.247)	0.234	0.940 (0.539, 1.638)	0.826	−0.075 (−0.213, 0.062)	0.282	−0.525 (−1.607, 0.556)	0.341
	exposed	0	1.00 (Ref)		1.00 (Ref)		1.00 (Ref)		1.00 (Ref)		1.00 (Ref)	
	exposed	1	1.219 (0.697, 2.132)	0.487	0.583 (0.312, 1.089)	0.090	1.629 (0.949, 2.797)	0.077	0.171 (0.006, 0.335)	0.042 *	−0.463 (−1.580, 0.654)	0.416
rs9470794	unexposed	0	1.00 (Ref)		1.00 (Ref)		1.00 (Ref)		1.00 (Ref)		1.00 (Ref)	
	unexposed	1	1.518 (0.545, 4.229)	0.425	1.393 (0.488, 3.977)	0.536	0.514 (0.239, 1.108)	0.090	0.086 (−0.148, 0.319)	0.472	−1.468 (−3.287, 0.351)	0.114
	exposed	0	1.00 (Ref)		1.00 (Ref)		1.00 (Ref)		1.00 (Ref)		1.00 (Ref)	
	exposed	1	1.417 (0.540, 3.715)	0.479	7.902 (1.063, 58.735)	0.043 *	1.709 (0.598, 4.883)	0.317	0.235 (−0.020, 0.490)	0.071	2.105 (0.363, 3.848)	0.018 *

* *p* < 0.05; †: No result due to complete separate data.

## Supplementary Materials

The following are available online at https://www.mdpi.com/2072-6643/12/12/3880/s1, Table S1: Hardy–Weinberg equilibrium test.
